# Supplementary material for: The Cambridge Questionnaire for Apathy and Impulsivity Traits (CamQUAIT): a novel assessment tool for frontotemporal lobar degeneration-related syndromes
Source: PLoS One. 2026 Apr 22;21(4):e0345545. doi: 10.1371/journal.pone.0345545 (PMC13102211; doi:10.1371/journal.pone.0345545)
Supplement: S1 File — (PDF) [file pone.0345545.s001.pdf]

S1 File. Final item set and scoring sheet for the Cambridge Questionnaire for Apathy and Impulsivity Traits (CamQUAIT).

Your Name: ....Date:..................................

Patient's Name:...

Relationship to Patient:...

Please answer the following questions about behaviour you have seen in recent weeks.

Circle the word that best applies (never, sometimes, often, always) for every question.

**The patient named above:**	**Never**	**Sometimes**	**Often**	**Always**	M	C
1. feels it is important to finish a job/task	Never	Sometimes	Often	Always		
2. is uncooperative	Never	Sometimes	Often	Always		
3. shows initiative	Never	Sometimes	Often	Always		
4. has outbursts of aggression (or temper)	Never	Sometimes	Often	Always		
5. likes to get things done during the day	Never	Sometimes	Often	Always		
6. is socially embarrassing	Never	Sometimes	Often	Always		
7. is impulsive (acts without thinking)	Never	Sometimes	Often	Always		
8. shows interest in things	Never	Sometimes	Often	Always		
9. is easily irritated	Never	Sometimes	Often	Always		
10. makes insensitive or tactless comments	Never	Sometimes	Often	Always		
11. is considerate of other family members and their concerns	Never	Sometimes	Often	Always		
12. can manage each day without being told what to do	Never	Sometimes	Often	Always		
13. likes to learn new things	Never	Sometimes	Often	Always		
14. gets excited when something good happens to him/her	Never	Sometimes	Often	Always		
15. is interested in meeting friends	Never	Sometimes	Often	Always		

SCORING

All items marked as part of the 'Motivation and Support' subscale are reverse scored. Subscores are indicated by the shaded boxes to the right of the scale.

Items are scored as follows*:

0 = Never

1 = Sometimes

2 = Often

3 = Always

*For reverse scored items, 3 = Never, 2 = Sometimes, 1 = Often, 0 = Always.

Subscores:

- Motivation and Support Sub-score (9 items):....../27
- Impulsivity and Challenging Behaviours Sub-score (6 items):...../18
- Total score (for clinical purposes only):...../45

Higher scores indicate greater impairment, reflecting increased behavioural change including apathy/impulsivity.
